# Neo-adjuvant radiation therapy provides a survival advantage in T3-T4 nodal positive gastric and gastroesophageal junction adenocarcinoma: a SEER database analysis

**DOI:** 10.1186/s12885-021-08534-9

**Published:** 2021-07-03

**Authors:** Yu-Jie Zhou, Xiao-Fan Lu, Jia-Lin Meng, Xin-Yuan Wang, Qing-Wei Zhang, Jin-Nan Chen, Qi-Wen Wang, Fang-Rong Yan, Xiao-Bo Li

**Affiliations:** 1 Division of Gastroenterology and Hepatology, Key Laboratory of Gastroenterology and Hepatology, Ministry of Health, Shanghai Institute of Digestive Disease, Renji Hospital, School of Medicine, Shanghai Jiao Tong University, 160 Pujian Road, Shanghai, 200127 China; 2 State Key Laboratory of Natural Medicines, Research Center of Biostatistics and Computational Pharmacy, China Pharmaceutical University, Nanjing, China; 3 Department of Urology, The First Affiliated Hospital of Anhui Medical University, Hefei, China

**Keywords:** Radiotherapy, Preoperative, Survival, Gastric cancer

## Abstract

**Background:**

Due to negative results in clinical trials of postoperative chemoradiation for gastric cancer, at present, there is a tendency to move chemoradiation therapy forward in gastric and gastroesophageal junction (GEJ) adenocarcinoma. Several randomized controlled trials (RCTs) are currently recruiting subjects to investigate the effect of neo-adjuvant radiotherapy (NRT) in gastric and GEJ cancer. Large retrospective studies may be beneficial in clarifying the potential benefit of NRT, providing implications for RCTs.

**Methods:**

We retrieved the clinicopathological and treatment data of gastric and GEJ adenocarcinoma patients who underwent surgical resection and chemotherapy between 2004 and 2015 from Surveillance, Epidemiology, and End Results (SEER) database. We compared survival between NRT and non-NRT patients among four clinical subgroups (T_1–2_N^−^, T_1–2_N^+^, T_3–4_N^−^, and T_3–4_N^+^).

**Results:**

Overall, 5272 patients were identified, among which 1984 patients received NRT. After adjusting confounding variables, significantly improved survival between patients with and without NRT was only observed in T_3–4_N^+^ subgroup [hazard ratio (HR) 0.79, 95% confidence interval (CI): 0.66–0.95; *P* = 0.01]. Besides, Kaplan-Meier plots showed significant cause-specific survival advantage of NRT in intestinal type (*P* <  0.001), but not in diffuse type (*P* = 0.11) for T_3–4_N^+^ patients. In the multivariate competing risk model, NRT still showed survival advantage only in T_3–4_ N^+^ patients (subdistribution HR: 0.77; 95% CI: 0.64–0.93; *P* = 0.006), but not in other subgroups.

**Conclusions:**

NRT might benefit resectable gastric and GEJ cancer patients of T3–4 stages with positive lymph nodes, particularly for intestinal-type. Nevertheless, these results should be interpreted with caution, and more data from ongoing RCTs are warranted.

**Supplementary Information:**

The online version contains supplementary material available at 10.1186/s12885-021-08534-9.

## Background

Radiation therapy (RT) has gained increasing attention in adjuvant treatment of resectable gastric cancer or gastroesophageal junction (GEJ) adenocarcinoma in the past two decades, since the landmark study of INT-0116 in US [1]. Subgroup analysis of INT-0116 trial indicated that male patients and patients with intestinal type of Lauren classification were more likely to benefit from adjuvant RT [2]. In Asian population, ARTIST trial targeting patients after D2 lymph node dissection showed negative results; but subgroup analysis implicated that adjuvant RT could potentially benefit a subset of patients with nodal involvement or intestinal histology type [3]. However, newly-released negative results of following ARTIST-II trial put the role of RT after R0 resection or D2 lymph node dissection into an awkward position [4]. Moreover, the CRITICS study also concluded that postoperative chemoradiation failed to improve survival rates compared with adjuvant chemotherapy in patients with resectable gastric cancer [5].

Due to abovementioned negative findings of research on postoperative chemoradiation, focus of RT in gastric cancer gradually turned to efficacy of preoperative chemoradiation. Advantages of preoperative RT include the potential for downstaging of gastric cancer with an elevated probability of R0 resection, and better tolerability [6, 7]. Recently, several neo-adjuvant treatments for gastric cancer have been evaluated in phase II and III randomized controlled trials (RCTs). CRITICS-II [8] and TOPGEAR [9] studies are actively investigating the effects of neo-adjuvant radiotherapy (NRT) in patients with stomach adenocarcinoma; the safety of preoperative chemoradiation has been proven according to interim analysis of preliminary results from TOPGEAR trial [10]. In China, two phase III RCTs, PREACT study [11] and NEO-CRAG (registration number NCT01815853), are currently underway to provide more evidence for efficacy of preoperative chemoradiation compared with preoperative chemotherapy in locally advanced gastric and GEJ adenocarcinoma.

To add to limited data from RCTs and facilitate evidence-based clinical decision on the use of preoperative RT in gastric and GEJ adenocarcinoma, we aimed to explore whether a subgroup of patients who have received chemotherapy and surgical treatment can gain additional survival benefit from NRT from a large population-based cancer registry database.

## Methods

### Study population

We retrieved the clinicopathological and treatment data of gastric and GEJ cancer patients diagnosed between 2004 and 2015 from National Cancer Institute’s Surveillance, Epidemiology, and End Results (SEER) database (1975–2016), released April 2019. The SEER database consists of 18 population-based cancer registries and covers about 28% of all US cancer cases [12]. Pathologic tumor stage was reevaluated according to American Joint Committee on Cancer (AJCC) 8th TNM staging system of gastric cancer [13]. The inclusion criteria were as follows: (1) the first primary tumor; (2) histologically confirmed gastric and GEJ adenocarcinoma; (3) surgery performed; (4) underwent chemotherapy before and/or after radical surgery. We excluded patients if (1) distant metastasis occurred; (2) T stage was unknown; (3) data of lymph node (LN) metastasis status were missing; (4) received postoperative radiotherapy; (5) lost to follow up within 30 days after surgery.

### Variables and outcomes

We histologically classified gastric adenocarcinoma according to Lauren classification: intestinal type was defined as 8144/3 (adenocarcinoma, intestinal type), 8140/3 (adenocarcinoma, not otherwise specified), 8010/3 (carcinoma, not otherwise specified), or 8211/3 (tubular adenocarcinoma); diffuse subtype was defined as 8145/3 (carcinoma, diffuse type), 8490/3 (signet ring cell carcinoma), or 8142/3 (linitis plastica), based on codes of International Classification of Disease for Oncology, 3rd edition (ICD-O-3) [14, 15].

Survival curves were plotted via the Kaplan-Meier method, and log-rank test was employed to determine significant overall survival (OS) and cause-specific survival (CSS) differences between patients with and without neo-adjuvant RT. Multivariate Cox regression analysis for OS was used to assess prognostic effects of preoperative RT, age, sex, tumor grade, tumor size, number of LN examined, Lauren classification, and surgery type in predefined subgroups of gastric cancer patients. In this study, CSS was defined as time from surgery to death from gastric and GEJ adenocarcinoma, and CSS information was unavailable in a few subjects. Subgroup analysis were performed in patients with different T and N stages and Lauren classification. The effect of NRT on OS and CSS in gastric cancer of intestinal and diffuse type was assessed by hazard ratios (HRs) with 95% confidence intervals (CIs).

### Statistical analysis

Continuous variables were presented as mean ± standard deviation, and categorical data were presented as numbers (percentage). Continuous variables with or without normal distribution were compared using the Student’s *t*-test or non-parametric Mann-Whitney U test, as appropriate. Categorical data were compared using the chi-squared test or the Fisher’s exact test, as appropriate. We employed proportional subdistribution hazards modeling (Fine and Gray’s competing risk regression model), an alternative to Cox regression when considering competing events [16], to assess combined effects of the variables on gastric cancer specific-survival, with results presented by subdistribution hazard ratio (SHR) and 95% CI.

All statistical analyses were performed by R version 3.6.0 (https://www.r-project.org/). For all statistical tests, a two-sided *P* value less than 0.05 was regarded statistically significant.

## Results

### Baseline characteristics of patients

We evaluated 5272 gastric and GEJ adenocarcinoma patients who underwent surgical resection and chemotherapy. The average age was 61.8 ± 11.6 years old, and 3761 (71.3%) were male. Among them, 1984 (37.6%) patients received NRT. Factors associated with utilization of NRT included younger age at diagnosis, male sex, diagnosed after year 2010, location of cardia, intestinal subtype of Lauren classification, and examined lymph nodes less than 15 (Table 1).

**Table 1:** Clinical and pathological features in patients with gastric and gastroesophageal junction adenocarcinoma, stratified by receipt of preoperative radiation therapy

Variables	No NRT	NRT	*P*
***N***	3288 (62.4%)	1984 (37.6%)	
Age (years)	62.0 ± 12.4	61.4 ± 10.2	0.01
Male (%)	2106 (64.1%)	1655 (83.4%)	< 0.001
Year of diagnosis			< 0.001
2004–2009	1321 (40.2%)	646 (32.6%)	
2010–2015	1967 (59.8%)	1338 (67.4%)	
Race/Ethnicity			< 0.001
Non-Hispanic White	1487 (45.2%)	1637 (82.5%)	
Black	431 (13.1%)	73 (3.7%)	
Hispanic White	692 (21.0%)	153 (7.7%)	
Asian/Pacific Islanders	618 (18.8%)	95 (4.8%)	
American Indian/Alaska Native	43 (1.3%)	25 (1.3%)	
Unknown	17 (0.5%)	1 (0.1%)	
Tumor differentiation			< 0.001
Well differentiated	71 (2.2%)	86 (4.3%)	
Moderately differentiated	659 (20.0%)	649 (32.7%)	
Poorly differentiated	2323 (70.7%)	1007 (50.8%)	
Undifferentiated	59 (1.8%)	37 (1.9%)	
Unknown	176 (5.4%)	205 (10.3%)	
Tumor size			< 0.001
≤ 3 cm	755 (23.0%)	534 (26.9%)	
3.1–5 cm	808 (24.6%)	552 (27.8%)	
> 5 cm	1273 (38.7%)	422 (21.3%)	
Unknown	452 (13.7%)	476 (24.0%)	
Location			< 0.001
Cardia	783 (23.8%)	1833 (92.4%)	
Fundus	118 (3.6%)	8 (0.4%)	
Body	343 (10.4%)	24 (1.2%)	
Antrum	753 (22.9%)	32 (1.6%)	
Pylorus	124 (3.8%)	5 (0.3%)	
Less curvature	383 (11.6%)	33 (1.7%)	
Greater curvature	145 (4.4%)	9 (0.5%)	
Overlapping/NOS	639 (19.4%)	40 (2.0%)	
Lauren classification			< 0.001
Intestinal	1988 (60.5%)	1607 (81.0%)	
Diffuse	1083 (32.9%)	271 (13.7%)	
Unclassified	217 (6.6%)	106 (5.3%)	
No. of LNs examined			< 0.001
< 15	1225 (37.3%)	1065 (53.7%)	
≥ 15	2063 (62.7%)	919 (46.3%)	
Pathologic T stage			< 0.001
T1	357 (10.9%)	195 (9.8%)	
T2	1762 (53.6%)	1116 (56.3%)	
T3	854 (26.0%)	590 (29.7%)	
T4	315 (9.6%)	83 (4.2%)	
Pathologic N stage			< 0.001
N0	897 (27.3%)	643 (32.4%)	
N1	1459 (44.4%)	1129 (56.9%)	
N2	629 (19.1%)	183 (9.2%)	
N3	303 (9.2%)	29 (1.5%)	
AJCC 8th TNM stage			< 0.001
I	835 (25.4%)	543 (27.4%)	
II	1661 (50.5%)	1265 (63.8%)	
III	792 (24.1%)	176 (8.9%)	
Surgery type			< 0.001
Partial gastrectomy	1980 (60.2%)	1150 (58.0%)	
Near total/total gastrectomy	1091 (33.2%)	409 (20.6%)	
Gastrectomy, NOS	217 (6.69%)	425 (21.4%)	

Cohort size, *n* = 5272. Categorical values are shown as n (%). Continuous variables are shown as mean ± standard deviation

### Survival benefit of NRT in patients with advanced stages and intestinal subtype

**Fig. 1:**
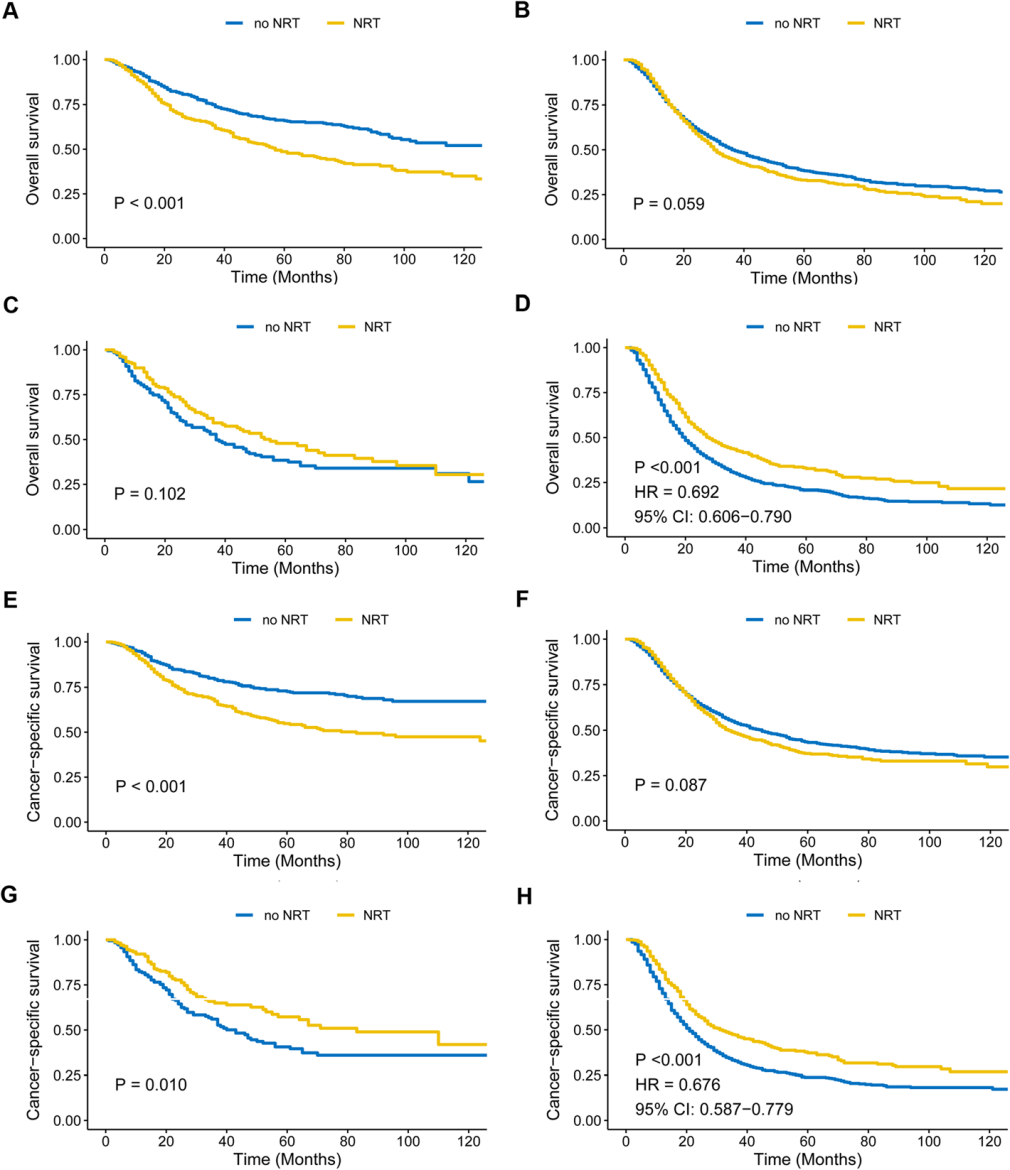
Overall survival (OS) and cause-specific survival (CSS) between NRT and no NRT patients, stratified by T and N stages. **A-D** Kaplan-Meier curves for OS in T_1–2_N^−^ (**A**), T_1–2_N^+^ (**B**), T_3–4_N^−^ (**C**), and T_3–4_N^+^ (**D**) clinical subgroups; **E-H** Kaplan-Meier curves for CSS in T_1–2_N^−^ (**E**), T_1–2_N^+^ (**F**), T_3–4_N^−^ (**G**), and T_3–4_N^+^ (**H**) clinical subgroups. CSS information was missing in a few patients. NRT: neo-adjuvant radiotherapy

Over a median follow-up of 59 months (interquartile range: 32–94 months), the 5-year OS and CSS for the entire cohort were 38.7 and 43.5%, respectively. In the entire cohort, NRT was not associated with improved OS or CSS (*P* = 0.51 and 0.29, respectively; Supplementary Fig. 1). Considering nodal status and tumor stage can influence the benefit of NRT in gastric cancer patients [1], we then divided the cohort into four subgroups to perform subgroup analysis: T_1–2_N^−^, T_1–2_N^+^, T_3–4_N^−^, and T_3–4_N^+^ (Fig. 1). Interestingly, NRT was shown to significantly increase both OS and CSS (both *P* <  0.001) only in nodal positive patients with pathologic T3-T4 stages (T_3–4_N^+^), and was associated with improved CSS but not OS in T_3–4_N^−^ patients (*P* = 0.01 and 0.10, respectively). For patients within T_1–2_N^+^ subgroup, no significantly different OS and CSS rates were observed between NRT and no NRT groups (*P* = 0.06 and 0.09, respectively). NRT even decreased OS and CSS in T_1–2_N^−^ subgroup (both *P* <  0.001).

**Fig. 2:**
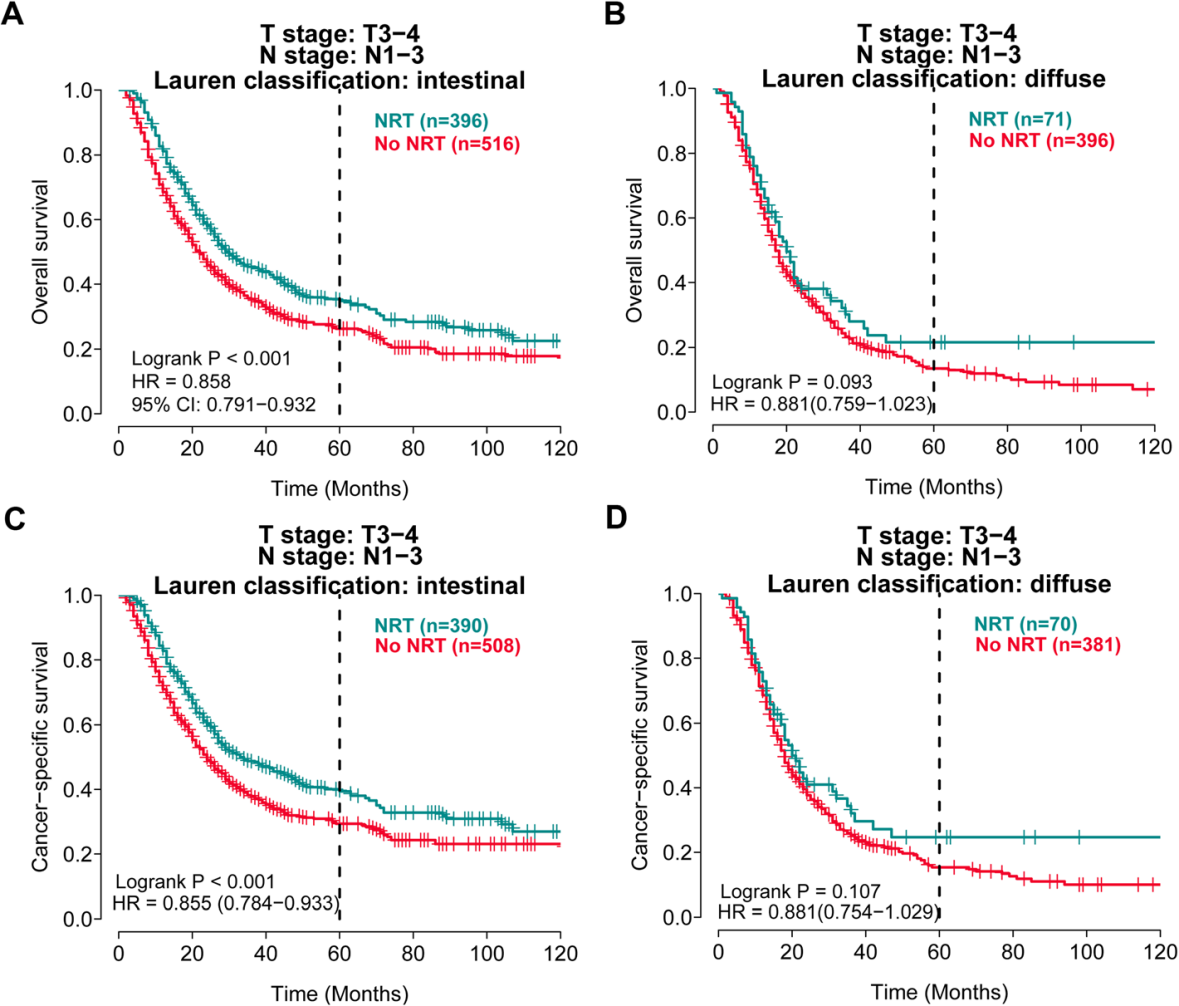
NRT was associated with improved overall survival (OS) and cause-specific survival (CSS) in gastric cancer with intestinal type of Lauren classification in T_3–4_N^+^ patients. **A-B** Kaplan-Meier curves for OS in intestinal type (**A**) and diffuse type (**B**); **C-D** Kaplan-Meier curves for CSS in intestinal type (**C**) and diffuse type (**D**)

Since a previous study reported that patients with intestinal type of Lauren classification were more likely to benefit from adjuvant RT in advanced GC [2], we also explored whether there was a survival difference between NRT and no NRT groups based on Lauren classification in T_3–4_N^+^ patients (Fig. 2). Kaplan-Meier plots showed survival advantage of NRT in both OS and CSS for intestinal type (both log-rank *P* <  0.001), but NRT was not shown to benefit either OS or CSS in diffuse type (log-rank *P* = 0.09 and 0.11, respectively).

### Multivariate cox analyses for OS and CSS among different subgroups, stratified by T stage and N stage

To adjust for confounding bias caused by unbalanced baseline variables, we employed multivariate Cox regression to examine the prognostic effect of NRT in four above-mentioned subgroups. We adjusted for known confounding variables that showed significantly difference between two groups in Table 1, including age, sex, race, year of diagnosis, tumor size, tumor differentiation, location, lymph nodes harvested, surgery type, tumor stage, and Lauren classification. The results for OS in T_1–2_N^−^, T_1–2_N^+^, T_3–4_N^−^, and T_3–4_N^+^ subgroups were illustrated in Table 2 and Supplementary Tables 1, 2, 3 and 4. We found NRT was associated with an improved OS only in T_3–4_N^+^ patients (adjusted HR: 0.79, 95% CI: 0.66–0.95; *P* = 0.01), and NRT was not linked with increased OS in T_3–4_N^−^ subgroup (adjusted HR: 0.76, 95% CI: 0.50–1.17; *P* = 0.22) after adjusting for known confounding variables.

**Table 2:** Results of multivariate Cox analysis for overall survival and cause-specific survival in different subgroups

Variable	N	Overall survival			Cause-specific survival		
		HR	95% CI	***P***	HR	95% CI	***P***
T_1–2_N^−^ subgroup							
RT							
no NRT	712	Ref.			Ref.		
NRT	474	1.28	0.98–1.67	0.073	1.32	0.98–1.8	0.071
Lauren classification							
Intestinal	852	Ref.			Ref.		
Diffuse	267	1.03	0.8–1.34	0.815	1.01	0.75–1.35	0.958
Unclassified	67	0.82	0.55–1.23	0.34	0.69	0.42–1.14	0.146
T stage							
T1	339	Ref.			Ref.		
T2	847	1.41	1.13–1.76	0.002	1.68	1.3–2.18	< 0.001
T_1–2_N^+^ subgroup							
RT							
no NRT	1407	Ref.			Ref.		
NRT	837	1.29	1.11–1.5	< 0.001	1.29	1.1–1.52	0.002
Lauren classification							
Intestinal	1579	Ref.			Ref.		
Diffuse	538	1.24	1.08–1.43	0.002	1.27	1.1–1.48	0.001
Unclassified	127	0.92	0.71–1.17	0.484	0.99	0.76–1.28	0.946
T stage							
T1	213	Ref.			Ref.		
T2	2031	1.28	1.04–1.57	0.022	1.42	1.11–1.8	0.005
N stage							
N1	1680	Ref.			Ref.		
N2	422	1.73	1.51–1.99	< 0.001	1.84	1.59–2.13	< 0.001
N3	142	2.27	1.83–2.82	< 0.001	2.32	1.83–2.93	< 0.001
T_3–4_N^−^ subgroup							
RT							
no NRT	185	Ref.					
NRT	169	0.76	0.5–1.17	0.216	0.71	0.44–1.14	0.157
Lauren classification							
Intestinal	252	Ref.					
Diffuse	82	1.4	0.97–2.02	0.074	1.42	0.96–2.09	0.08
Unclassified	20	0.62	0.29–1.32	0.213	0.71	0.31–1.59	0.4
T stage							
T3	272	Ref.					
T4	82	2.28	1.52–3.41	< 0.001	2.29	1.49–3.51	< 0.001
T_3–4_N^+^ subgroup							
RT							
no NRT	984	Ref.					
NRT	504	0.79	0.66–0.95	0.01	0.75	0.62–0.92	0.004
Lauren classification							
Intestinal	912	Ref.					
Diffuse	467	1.19	1.02–1.38	0.023	1.2	1.02–1.41	0.024
Unclassified	109	0.99	0.77–1.26	0.92	0.89	0.68–1.17	0.413
T stage							
T3	1172	Ref.					
T4	316	1.23	1.06–1.42	0.008	1.26	1.07–1.47	0.004
N stage							
N1	908	Ref.					
N2	390	1.66	1.43–1.92	< 0.001	1.63	1.39–1.91	< 0.001
N3	190	2.07	1.69–2.54	< 0.001	2.17	1.75–2.69	< 0.001

Models for T_1–2_N^−^ and T_3–4_N^−^ subgroups: adjusted for RT, age, sex, race, diagnostic time, tumor size, tumor differentiation, tumor site, number of lymph node examined, surgery type, Lauren classification, and T stage

Models for T_1–2_N^+^ and T_3–4_N^+^ subgroups: adjusted for adjusted for RT, age, sex, race, diagnostic time, tumor size, tumor differentiation, tumor site, number of lymph node examined, surgery type, Lauren classification, T stage, and N stage. The detailed results were shown in Supplementary Tables 1, 2, 3 and 4

*RT* radiation therapy, *NRT* neo-adjuvant radiotherapy, *NOS* not otherwise specific. Data are presented as hazard ratios (HRs) and 95% confidence intervals (CIs) measured by multivariable Cox regression analyses, with overall survival and cause-specific survival as the outcome, respectively

Similar results were shown for CSS in four clinical subgroups. NRT was associated with a significantly improved CSS only in T_3–4_N^+^ patients (adjusted HR: 0.75, 95% CI: 0.62–0.92; *P* = 0.004; Table 2 and Supplementary Table 4). Moreover, receipt of NRT did not show gastric cancer-specific survival benefit in either T_1–2_N^−^ (adjusted HR: 1.32, 95% CI: 0.98–1.80; *P* = 0.07), T_1–2_N^+^ subgroup (adjusted HR: 1.29, 95% CI: 1.10–1.52; *P* = 0.002), or T_3–4_N^−^ patients (adjusted HR: 0.71, 95% CI: 0.44–1.14; *P* = 0.16; Table 2).

### Competing risk model showed survival benefit of NRT in locally advanced (T_3–4_ N^+^) patients

**Fig. 3:**
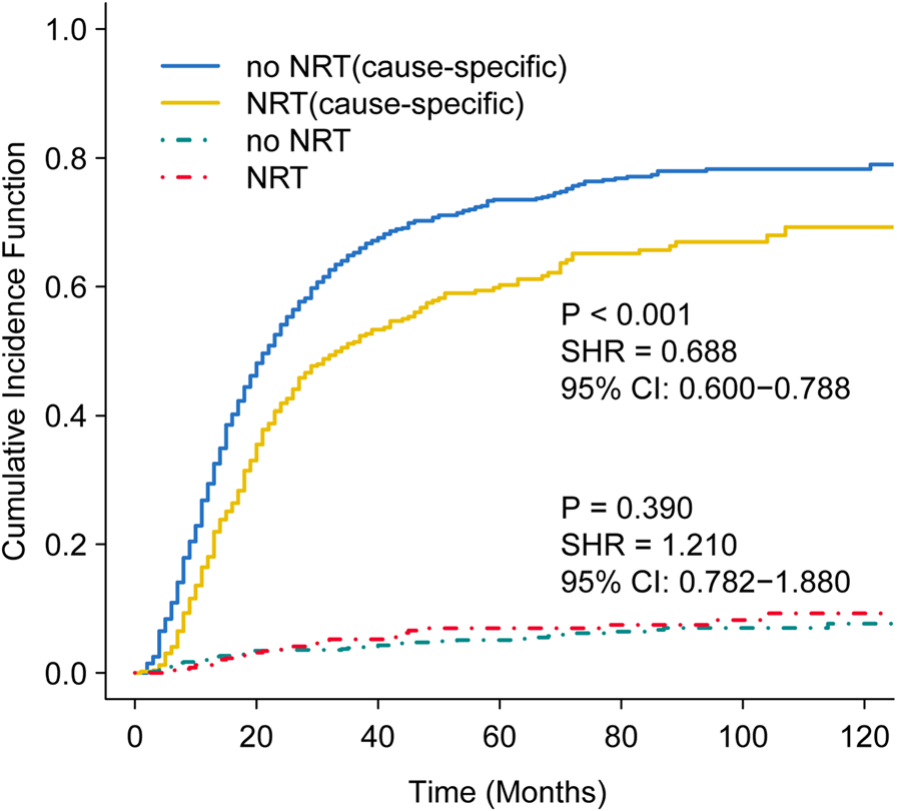
Cumulative incidence estimates of death stratified by administration of NRT in node-positive T3-T4 gastric cancer patients (solid line: gastric cancer-specific death; dotted line: other cause of death). NRT: neo-adjuvant radiotherapy

Cumulative incidence curve by administration of NRT in locally advanced patients was illustrated in Fig. 3, taking into account other causes of death as competing risk (dotted lines). Cumulative incidence function (CIF) decreased in T_3–4_ N^+^ patients receiving NRT for cause-specific death (SHR: 0.69; 95% CI: 0.60–0.79; *P* <  0.001), and CIF did not show significant difference between NRT and no NRT groups for other causes of death (SHR: 1.21; 95% CI: 0.78–1.88; *P* = 0.39).

Taking deaths not related to gastric cancer and confounding bias into consideration, we also performed multivariate Fine and Gray’s proportional subdistribution hazards modeling, in which deaths not related to gastric cancer was regarded as competing risks. As shown in Supplementary Figs. 2, 3, 4 and 5, NRT still showed survival advantage only in locally advanced (T_3–4_ N^+^) patients (SHR: 0.77; 95% CI: 0.64–0.93; *P* = 0.006), but not in T_1–2_N^−^ (SHR: 1.33; 95% CI: 0.96–1.85; *P* = 0.09), T_1–2_N^+^ (SHR: 1.26; 95% CI: 1.07–1.47; *P* = 0.005), or T_3–4_N^−^ subgroup (SHR: 0.67; 95% CI: 0.42–1.08; *P* = 0.10). In both univariate and multivariate competing risk models, receipt of NRT was associated with improved gastric cancer-specific survival in locally advanced (T_3–4_ N^+^) patients.

## Discussion

In this large cross-sectional study to investigate the association between NRT and prognosis of gastric and GEJ adenocarcinoma, we found a significant association between receipt of NRT and prolonged survival only in pathologic T3-T4 patients with nodal involvement. We have employed multivariate Cox regression and competing risk model to reduce confounding bias caused by being retrospective in nature, and our findings remained valid in these multivariate models.

Interestingly, for other clinical subgroups, administration of NRT failed to provide an additional benefit in these patients who have already undergone chemotherapy. In some earlier clinical stages, receipt of NRT even associated with reduced survival, suggesting that it might be prudent to give NRT in these patients. For the time being, long-term outcomes between NRT and no NRT groups have been elucidated only in RCT for esophageal cancer patients [17], survival benefit of NRT for at least a part of gastric cancer patients remain pending validation in prospective studies. In reality, although NRT for gastric cancer was not widely applied in clinical practice, patients included in our study might receive NRT for the following reasons: 1) to increase the rate of R0 resection in T3-T4 tumors; 2) compared with postoperative patients, preoperative patients have better tolerance to radiotherapy; 3) in some tertiary hospitals, RT is readily available [18].

Our findings were not consistent with all previous retrospective findings, mainly due to subgroup analysis based on T and N stages exclusively performed in our study. Recently, a large-scale retrospective study (*n* = 1048) based on National Cancer Database showed preoperative radiotherapy contributed to a prolonged OS with a marginally significant *P* value (log-rank *P* = 0.04), compared with patients receiving perioperative chemotherapy [19]. A possible explanation was that in their study, 15.4% patients were T1–2 stages and 24% were node-negative, and they neither conducted subgroup analyses, nor compared CSS rates. Given the observation of no survival benefit, or even reduced survival of neo-adjuvant RT for T1–2 or N0 patients in our study, results of subgroup analysis in locally advanced subjects may yield a more pronounced result. Another study of SEER database by Shridhar et al. included 424 gastric cancer patients who underwent preoperative RT, and 115 of them were with T3–4 stages [20]. Shridhar et al. concluded that NRT was beneficial for node-positive patients, but they also failed to perform subgroup analysis stratified by T stages. Our results based on SEER indicated that T1–2 nodal positive subjects could not gain survival advantage from neo-adjuvant RT.

Previous RCTs have shown that patients with intestinal-type gastric cancer were more likely to gain survival benefit from postoperative RT [2, 3], but whether this association also exists in the context of NRT is unknown. We found Lauren classification could also predict survival benefit of preoperative RT. In locally advanced (T_3–4_ N^+^) patients receiving NRT, the intestinal type subgroup showed significantly prolonged survival (35.1 vs. 29.4 months of RMST, *P* <  0.001), but the subgroup of diffuse type did not. A possible explanation for the selectivity for intestinal type tumors might be that the diffuse type tumor was more frequently associated with undifferentiated gastric cancer and poor prognosis [21], and any association with NRT might be masked by the prognostic impact of its undifferentiated histology. The effect of NRT for patients with diffuse-type gastric cancer needs to be further explored in ongoing RCTs.

Several limitations need to be noted in our study. First, even though we tried to minimize selection bias by only including patients who have undergone chemotherapy, the information of the chemotherapy regimen, and type of chemotherapy (neo-adjuvant, adjuvant, or perioperative) was missing in SEER database. That is to say, the NRT group in this study incorporated patients who received either neoadjuvant chemoradiotherapy or neoadjuvant radiotherapy, which might be a potentially significant confounder in our study. Furthermore, detailed information on radiation dosing and RT toxicity was missing. Although some studies have proven tolerable toxicity of neo-adjuvant chemoradiation in most gastric and GEJ cancer patients [6, 10, 22], optimum dosing and side effects of preoperative RT need to be further clarified in RCTs. In addition, some alternative endpoints, such as local recurrence and radical resection rate (R0), could not be analyzed due to limited information recorded in SEER registry.

## Conclusions

In summary, our study suggested that addition of preoperative radiation to chemotherapy could provide a survival advantage in resectable gastric cancer patients of T3–4 stages with positive lymph nodes, particularly for patients with intestinal-type cancer. For T1–2 stages or node-negative patients, NRT might not result in survival benefit. Nevertheless, our results should be interpreted with caution, considering observation bias caused by it being retrospective in nature and more data from ongoing RCTs in assessing efficacy of preoperative RT in locally advanced gastric and GEJ adenocarcinoma are warranted.

## Supplementary Information

**Additional file 1: Supplementary Table 1.** Results of multivariate Cox analysis for overall survival and cause-specific survival in T_1–2_N^−^ subgroup. **Supplementary Table 2.** Results of multivariate Cox analysis for overall survival and cause-specific survival in T_1–2_N^+^ subgroup. **Supplementary Table 3.** Results of multivariate Cox analysis for overall survival and cause-specific survival in T_3–4_N^−^ subgroup. **Supplementary Table 4.** Results of multivariate Cox analysis for overall survival and cause-specific survival in T_3–4_N^+^ subgroup. **Supplementary Figure 1.** Kaplan-Meier curves for OS (A) and CSS (B), stratified by administration of NRT. OS: overall survival; CSS: cause-specific survival; NRT: neo-adjuvant radiotherapy. **Supplementary Figure 2.** Forest plot of competing risk model in T_1–2_N^−^ subgroup. RT: radiation therapy; NRT: neo-adjuvant radiotherapy; SHR: subdistribution hazard ratio; CI: confidence interval; NOS: not otherwise specific. **Supplementary Figure 3.** Forest plot of competing risk model in T_1–2_N^+^ subgroup. RT: radiation therapy; NRT: neo-adjuvant radiotherapy; SHR: subdistribution hazard ratio; CI: confidence interval; NOS: not otherwise specific. **Supplementary Figure 4.** Forest plot of competing risk model in T_3–4_N^−^ subgroup. RT: radiation therapy; NRT: neo-adjuvant radiotherapy; SHR: subdistribution hazard ratio; CI: confidence interval; NOS: not otherwise specific. **Supplementary Figure 5.** Forest plot of competing risk model in T_3–4_N^+^ subgroup. RT: radiation therapy; NRT: neo-adjuvant radiotherapy; SHR: subdistribution hazard ratio; CI: confidence interval; NOS: not otherwise specific.

## Data Availability

The datasets supporting the conclusions of this article are available in the SEER database.

## Abbreviations

RT: Radiation therapy
GEJ: Gastroesophageal junction
RCT: Randomized controlled trial
NRT: Neo-adjuvant radiotherapy
SEER: Surveillance, Epidemiology, and End Results
AJCC: American Joint Committee on Cancer
LN: Lymph node
ICD-O-3: International Classification of Disease for Oncology, 3rd edition
OS: Overall survival
CSS: Cause-specific survival
HR: Hazard ratio
CI: Confidence interval
CIF: Cumulative incidence function
SHR: Subdistribution hazard ratio
