# Supplementary material for: Neo-adjuvant radiation therapy provides a survival advantage in T3-T4 nodal positive gastric and gastroesophageal junction adenocarcinoma: a SEER database analysis
Source: BMC Cancer. 2021 Jul 3;21:771. doi: 10.1186/s12885-021-08534-9 (PMC8254219; doi:10.1186/s12885-021-08534-9)
Supplement: Supplementary file 1 — Additional file 1: Supplementary Table 1. Results of multivariate Cox analysis for overall survival and cause-specific survival in T1–2N− subgroup. Supplementary Table 2. Results of multivariate Cox analysis for overall survival and cause-specific survival in T1–2N+ subgroup. Supplementary Table 3. Results of multivariate Cox analysis for overall survival and cause-specific survival in T3–4N− subgroup. Supplementary Table 4. Results of multivariate Cox analysis for overall survival and cause-specific survival in T3–4N+ subgroup. Supplementary Figure 1. Kaplan-Meier curves for OS (A) and CSS (B), stratified by administration of NRT. OS: overall survival; CSS: cause-specific survival; NRT: neo-adjuvant radiotherapy. Supplementary Figure 2. Forest plot of competing risk model in T1–2N− subgroup. RT: radiation therapy; NRT: neo-adjuvant radiotherapy; SHR: subdistribution hazard ratio; CI: confidence interval; NOS: not otherwise specific. Supplementary Figure 3. Forest plot of competing risk model in T1–2N+ subgroup. RT: radiation therapy; NRT: neo-adjuvant radiotherapy; SHR: subdistribution hazard ratio; CI: confidence interval; NOS: not otherwise specific. Supplementary Figure 4. Forest plot of competing risk model in T3–4N− subgroup. RT: radiation therapy; NRT: neo-adjuvant radiotherapy; SHR: subdistribution hazard ratio; CI: confidence interval; NOS: not otherwise specific. Supplementary Figure 5. Forest plot of competing risk model in T3–4N+ subgroup. RT: radiation therapy; NRT: neo-adjuvant radiotherapy; SHR: subdistribution hazard ratio; CI: confidence interval; NOS: not otherwise specific. [file 12885_2021_8534_MOESM1_ESM.docx]

## Supplementary Tables

**Supplementary Table 1**. Results of multivariate Cox analysis for overall survival and cause-specific survival in T_1-2_N^-^ subgroup.

|  |  | **Overall survival** | | |  | **Cause-specific survival** | | |
| --- | --- | --- | --- | --- | --- | --- | --- | --- |
| **Variable** | **N** | **HR** | **95% CI** | ***P*** | | **HR** | **95% CI** | ***P*** |
| RT |  |  |  |  | |  |  |  |
| no NRT | 712 | Ref. |  |  | | Ref. |  |  |
| NRT | 474 | 1.28 | 0.98-1.67 | 0.073 | | 1.32 | 0.98-1.8 | 0.071 |
| Age |  |  |  |  | |  |  |  |
| Per 10 years  Sex  Female  Male  Race  White  Black  Others  Diagnostic time  2004-2009  2010-2015  Tumor size  ≤3cm  3.1-5cm  ＞5cm  Unknown  Tumor differentiation  Good/Moderate  Poor/Undifferentiated  Unknown  Site  Cardia  Fundus/Body  Antrum/Pylorus  Overlapping/NOS  Lymph node examined  ＜15  ≥15  Surgery  Partial gastrectomy  Near total/Total gastrectomy  Gastrectomy/NOS  Lauren classification  Intestinal  Diffuse  Unclassified  T stage  T1  T2 | 1186  338  848  886  110  190  405  781  435  252  218  281  422  643  121  595  260  208  123  634  552  741  293  152  852  267  67  339  847 | 1.25  Ref.  1.12  Ref.  1.1  0.77  Ref.  0.78  Ref.  0.8  1.31  1.19  Ref.  1.2  1.14  Ref.  0.71  0.75  1.18  Ref.  0.64  Ref.  1.17  1.49  Ref.  1.03  0.82  Ref.  1.41 | 1.14-1.37  0.89-1.4  0.77-1.57  0.56-1.06  0.63-0.95  0.61-1.06  1-1.72  0.93-1.52  0.96-1.5  0.81-1.6  0.5-0.99  0.52-1.09  0.81-1.73  0.52-0.78  0.93-1.48  1.15-1.94  0.8-1.34  0.55-1.23  1.13-1.76 | <0.001  0.342  0.601  0.106  0.013  0.118  0.05  0.16  0.101  0.457  0.044  0.129  0.39  <0.001  0.175  0.003  0.815  0.34  0.002 | | 1.13  Ref.  1  Ref.  1  0.8  Ref.  0.69  Ref.  0.81  1.5  1.28  Ref.  1.37  1.22  Ref.  0.58  0.7  1.21  Ref.  0.54  Ref.  1.24  1.63  Ref.  1.01  0.69  Ref.  1.68 | 1.02-1.26  0.78-1.3  0.65-1.52  0.55-1.16  0.55-1.16  0.59-1.12  1.11-2.04  0.97-1.69  1.06-1.77  0.83-1.8  0.39-0.87  0.46-1.07  0.79-1.86  0.43-0.69  0.96-1.62  1.22-2.19  0.75-1.35  0.42-1.14  1.3-2.18 | 0.015  0.974  0.984  0.239  0.001  0.196  0.009  0.087  0.016  0.303  0.009  0.103  0.384  <0.001  0.105  0.001  0.958  0.146  <0.001 |

RT: radiation therapy; NRT: neo-adjuvant radiotherapy; HR: hazard ratio; CI: confidence interval; NOS: not otherwise specific.

**Supplementary Table 2**. Results of multivariate Cox analysis for overall survival and cause-specific survival in T_1-2_N^+^ subgroup.

|  |  | **Overall survival** | | |  | **Cause-specific survival** | | |
| --- | --- | --- | --- | --- | --- | --- | --- | --- |
| **Variable** | **N** | **HR** | **95% CI** | ***P*** | | **HR** | **95% CI** | ***P*** |
| RT |  |  |  |  | |  |  |  |
| no NRT | 1407 | Ref. |  |  | | Ref. |  |  |
| NRT | 837 | 1.29 | 1.11-1.5 | <0.001 | | 1.29 | 1.1-1.52 | 0.002 |
| Age |  |  |  |  | |  |  |  |
| Per 10 years  Sex  Female  Male  Race  White  Black  Others  Diagnostic time  2004-2009  2010-2015  Tumor size  ≤3cm  3.1-5cm  ＞5cm  Unknown  Tumor differentiation  Good/Moderate  Poor/Undifferentiated  Unknown  Site  Cardia  Fundus/Body  Antrum/Pylorus  Overlapping/NOS  Lymph node examined  ＜15  ≥15  Surgery  Partial gastrectomy  Near total/Total gastrectomy  Gastrectomy/NOS  Lauren classification  Intestinal  Diffuse  Unclassified  T stage  T1  T2  N stage  N1  N2  N3 | 2244  600  1644  1680  231  333  807  1437  528  645  711  360  617  1478  149  1155  448  374  267  876  1368  1319  647  278  1579  538  127  213  2031  1680  422  142 | 1.17  Ref.  1.14  Ref.  0.98  0.76  Ref.  0.7  Ref.  0.97  1.15  1.02  Ref.  1.28  1.1  Ref.  0.89  1.08  1.02  Ref.  0.79  Ref.  1.21  1.2  Ref.  1.24  0.92  Ref.  1.28  Ref.  1.73  2.27 | 1.12-1.23  1-1.3  0.81-1.19  0.64-0.91  0.63-0.79  0.83-1.14  0.98-1.34  0.85-1.23  1.12-1.46  0.86-1.4  0.74-1.06  0.89-1.3  0.83-1.25  0.7-0.89  1.06-1.39  1.01-1.44  1.08-1.43  0.71-1.17  1.04-1.57  1.51-1.99  1.83-2.82 | <0.001  0.048  0.82  0.002  <0.001  0.708  0.086  0.799  <0.001  0.442  0.193  0.454  0.846  <0.001  0.004  0.043  0.002  0.484  0.022  <0.001  <0.001 | | 1.12  Ref.  1.2  Ref.  0.96  0.76  Ref.  0.68  Ref.  0.97  1.18  1.04  Ref.  1.37  1.11  Ref.  0.87  1.05  1.02  Ref.  0.78  Ref.  1.16  1.14  Ref.  1.27  0.99  Ref.  1.42  Ref.  1.84  2.32 | 1.06-1.18  1.04-1.39  0.77-1.18  0.63-0.92  0.6-0.77  0.82-1.16  0.99-1.4  0.85-1.28  1.18-1.59  0.84-1.45  0.71-1.06  0.85-1.3  0.82-1.27  0.69-0.88  1.01-1.34  0.94-1.39  1.1-1.48  0.76-1.28  1.11-1.8  1.59-2.13  1.83-2.93 | <0.001  0.015  0.673  0.005  <0.001  0.746  0.061  0.678  <0.001  0.467  0.162  0.647  0.863  <0.001  0.004  0.174  0.001  0.946  0.005  <0.001  <0.001 |

RT: radiation therapy; NRT: neo-adjuvant radiotherapy; HR: hazard ratio; CI: confidence interval; NOS: not otherwise specific.

**Supplementary Table 3**. Results of multivariate Cox analysis for overall survival and cause-specific survival in T_3-4_N^-^ subgroup.

|  |  | **Overall survival** | | |  | **Cause-specific survival** | | |
| --- | --- | --- | --- | --- | --- | --- | --- | --- |
| **Variable** | **N** | **HR** | **95% CI** | ***P*** | | **HR** | **95% CI** | ***P*** |
| RT |  |  |  |  | |  |  |  |
| no NRT | 185 | Ref. |  |  | |  |  |  |
| NRT | 169 | 0.76 | 0.5-1.17 | 0.216 | | 0.71 | 0.44-1.14 | 0.157 |
| Age |  |  |  |  | |  |  |  |
| Per 10 years  Sex  Female  Male  Race  White  Black  Others  Diagnostic time  2004-2009  2010-2015  Tumor size  ≤3cm  3.1-5cm  ＞5cm  Unknown  Tumor differentiation  Good/Moderate  Poor/Undifferentiated  Unknown  Site  Cardia  Fundus/Body  Antrum/Pylorus  Overlapping/NOS  Lymph node examined  ＜15  ≥15  Surgery  Partial gastrectomy  Near total/Total gastrectomy  Gastrectomy/NOS  Lauren classification  Intestinal  Diffuse  Unclassified  T stage  T3  T4 | 354  101  253  287  21  46  144  210  107  84  88  75  110  211  33  197  52  67  38  197  157  211  82  61  252  82  20  272  82 | 1.23  Ref.  1.26  Ref.  0.52  0.57  Ref.  0.78  Ref.  1.08  0.98  0.84  Ref.  1.36  1.04  Ref.  0.83  1.06  0.7  Ref.  0.73  Ref.  1.45  1.72  Ref.  1.4  0.62  Ref.  2.28 | 1.06-1.42  0.89-1.78  0.26-1.04  0.33-0.96  0.57-1.07  0.7-1.67  0.64-1.5  0.54-1.3  0.95-1.94  0.57-1.88  0.48-1.44  0.64-1.75  0.39-1.24  0.53-1  1-2.12  1.16-2.54  0.97-2.02  0.29-1.32  1.52-3.41 | 0.006  0.197  0.064  0.036  0.129  0.718  0.92  0.426  0.096  0.897  0.513  0.821  0.222  0.05  0.052  0.007  0.074  0.213  <0.001 | | 1.16  1.3  0.55  0.45  0.75  0.95  0.79  0.75  1.41  0.89  1.08  1.26  0.85  0.63  1.42  1.77  1.42  0.71  2.29 | 0.99-1.36  0.89-1.88  0.27-1.13  0.25-0.82  0.53-1.05  0.6-1.51  0.5-1.27  0.46-1.2  0.95-2.1  0.45-1.75  0.61-1.93  0.74-2.17  0.46-1.57  0.45-0.9  0.94-2.14  1.15-2.73  0.96-2.09  0.31-1.59  1.49-3.51 | 0.063  0.172  0.102  0.009  0.095  0.824  0.334  0.228  0.088  0.73  0.791  0.395  0.6  0.011  0.096  0.01  0.08  0.4  <0.001 |

RT: radiation therapy; NRT: neo-adjuvant radiotherapy; HR: hazard ratio; CI: confidence interval; NOS: not otherwise specific.

**Supplementary Table 4**. Results of multivariate Cox analysis for overall survival and cause-specific survival in T_3-4_N^+^ subgroup.

|  |  | **Overall survival** | | |  | **Cause-specific survival** | | |
| --- | --- | --- | --- | --- | --- | --- | --- | --- |
| **Variable** | **N** | **HR** | **95% CI** | ***P*** | | **HR** | **95% CI** | ***P*** |
| RT |  |  |  |  | |  |  |  |
| no NRT | 984 | Ref. |  |  | |  |  |  |
| NRT | 504 | 0.79 | 0.66-0.95 | 0.01 | | 0.75 | 0.62-0.92 | 0.004 |
| Age |  |  |  |  | |  |  |  |
| Per 10 years  Sex  Female  Male  Race  White  Black  Others  Diagnostic time  2004-2009  2010-2015  Tumor size  ≤3cm  3.1-5cm  ＞5cm  Unknown  Tumor differentiation  Good/Moderate  Poor/Undifferentiated  Unknown  Site  Cardia  Fundus/Body  Antrum/Pylorus  Overlapping/NOS  Lymph node examined  ＜15  ≥15  Surgery  Partial gastrectomy  Near total/Total gastrectomy  Gastrectomy/NOS  Lauren classification  Intestinal  Diffuse  Unclassified  T stage  T3  T4  N stage  N1  N2  N3 | 1488  472  1016  1116  142  230  611  877  219  379  678  212  316  1094  78  669  303  265  251  583  905  859  478  151  912  467  109  1172  316  908  390  190 | 1.07  Ref.  1.07  Ref.  0.93  1  Ref.  0.94  Ref.  1.12  1  1.1  Ref.  1.31  0.7  Ref.  0.99  0.94  1.12  Ref.  0.6  Ref.  1.19  1.08  Ref.  1.19  0.99  Ref.  1.23  Ref.  1.66  2.07 | 1.02-1.13  0.93-1.23  0.75-1.16  0.84-1.19  0.82-1.07  0.91-1.38  0.82-1.22  0.87-1.38  1.1-1.56  0.5-0.97  0.8-1.22  0.76-1.17  0.89-1.39  0.52-0.69  1.03-1.37  0.86-1.34  1.02-1.38  0.77-1.26  1.06-1.42  1.43-1.92  1.69-2.54 | 0.009  0.326  0.526  0.971  0.321  0.271  0.992  0.425  0.002  0.035  0.889  0.595  0.336  <0.001  0.021  0.52  0.023  0.92  0.008  <0.001  <0.001 | | 1.04  1.03  0.92  0.98  0.93  1.12  1.02  1.06  1.35  0.76  0.9  0.87  1.03  0.61  1.23  1.08  1.2  0.89  1.26  1.63  2.17 | 0.98-1.1  0.89-1.19  0.73-1.15  0.81-1.18  0.82-1.07  0.9-1.4  0.83-1.26  0.83-1.36  1.12-1.62  0.53-1.08  0.72-1.13  0.68-1.09  0.82-1.31  0.53-0.71  1.06-1.44  0.86-1.36  1.02-1.41  0.68-1.17  1.07-1.47  1.39-1.91  1.75-2.69 | 0.179  0.714  0.474  0.813  0.331  0.301  0.824  0.626  0.002  0.125  0.374  0.227  0.786  <0.001  0.007  0.522    0.024  0.413  0.004  <0.001  <0.001 |

RT: radiation therapy; NRT: neo-adjuvant radiotherapy; HR: hazard ratio; CI: confidence interval; NOS: not otherwise specific.

## Supplementary Figures


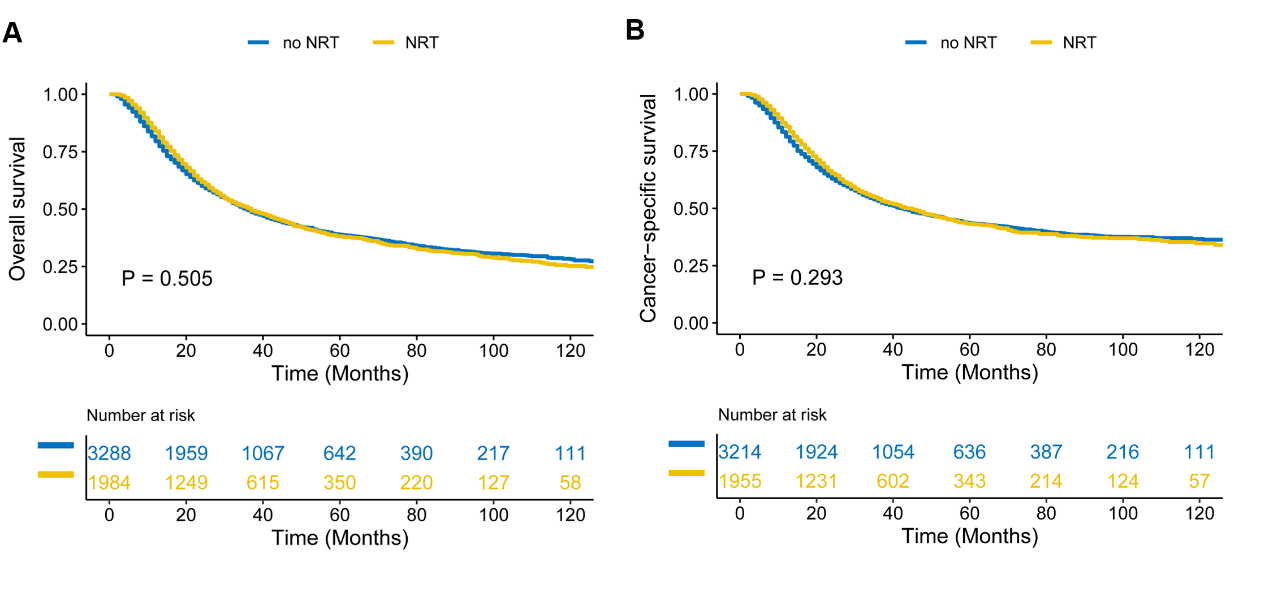


Supplementary Figure 1. Kaplan-Meier curves for OS (A) and CSS (B), stratified by administration of NRT.

OS: overall survival; CSS: cause-specific survival; NRT: neo-adjuvant radiotherapy.


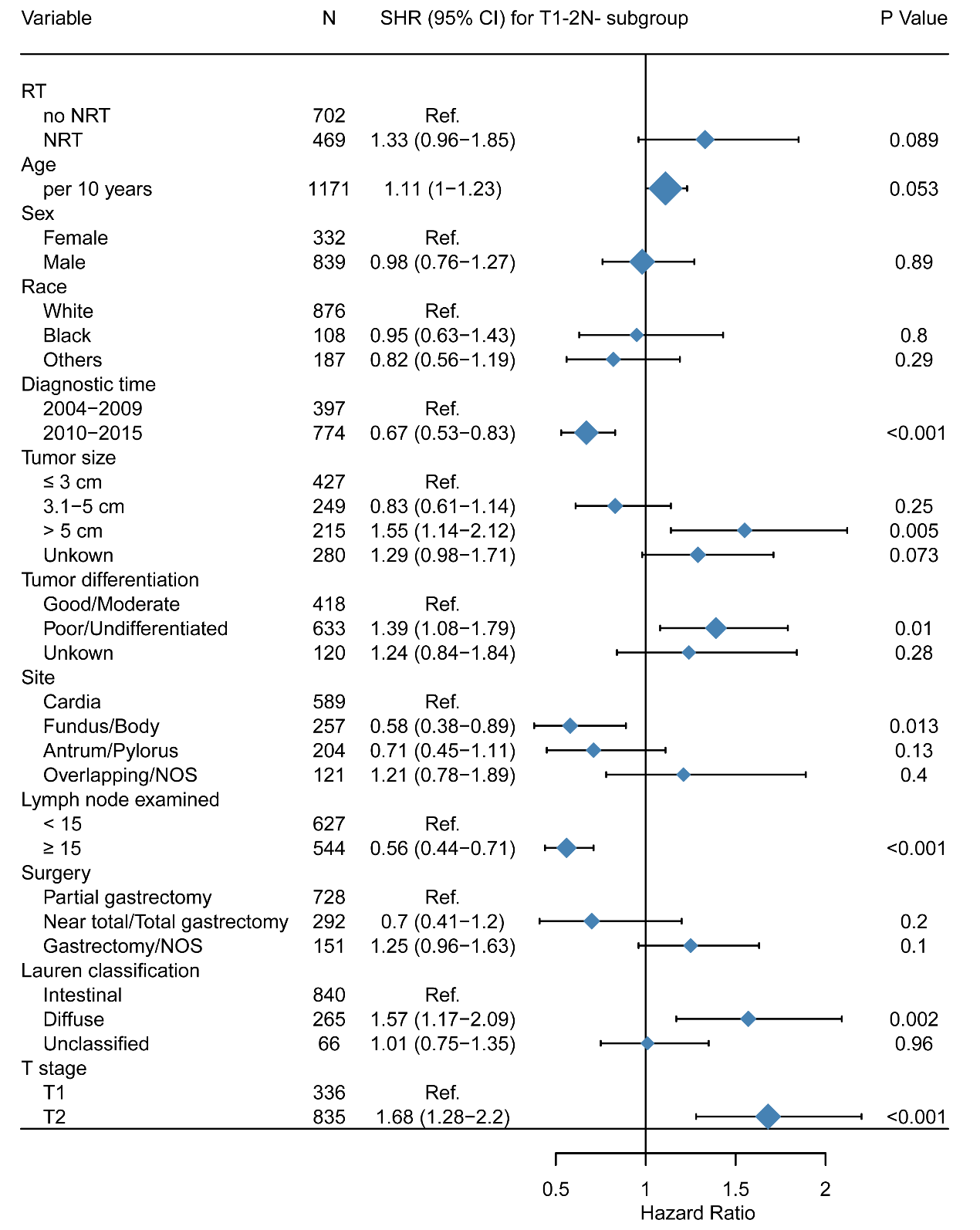


Supplementary Figure 2. Forest plot of competing risk model in T_1-2_N^-^ subgroup.

RT: radiation therapy; NRT: neo-adjuvant radiotherapy; SHR: subdistribution hazard ratio; CI: confidence interval; NOS: not otherwise specific.


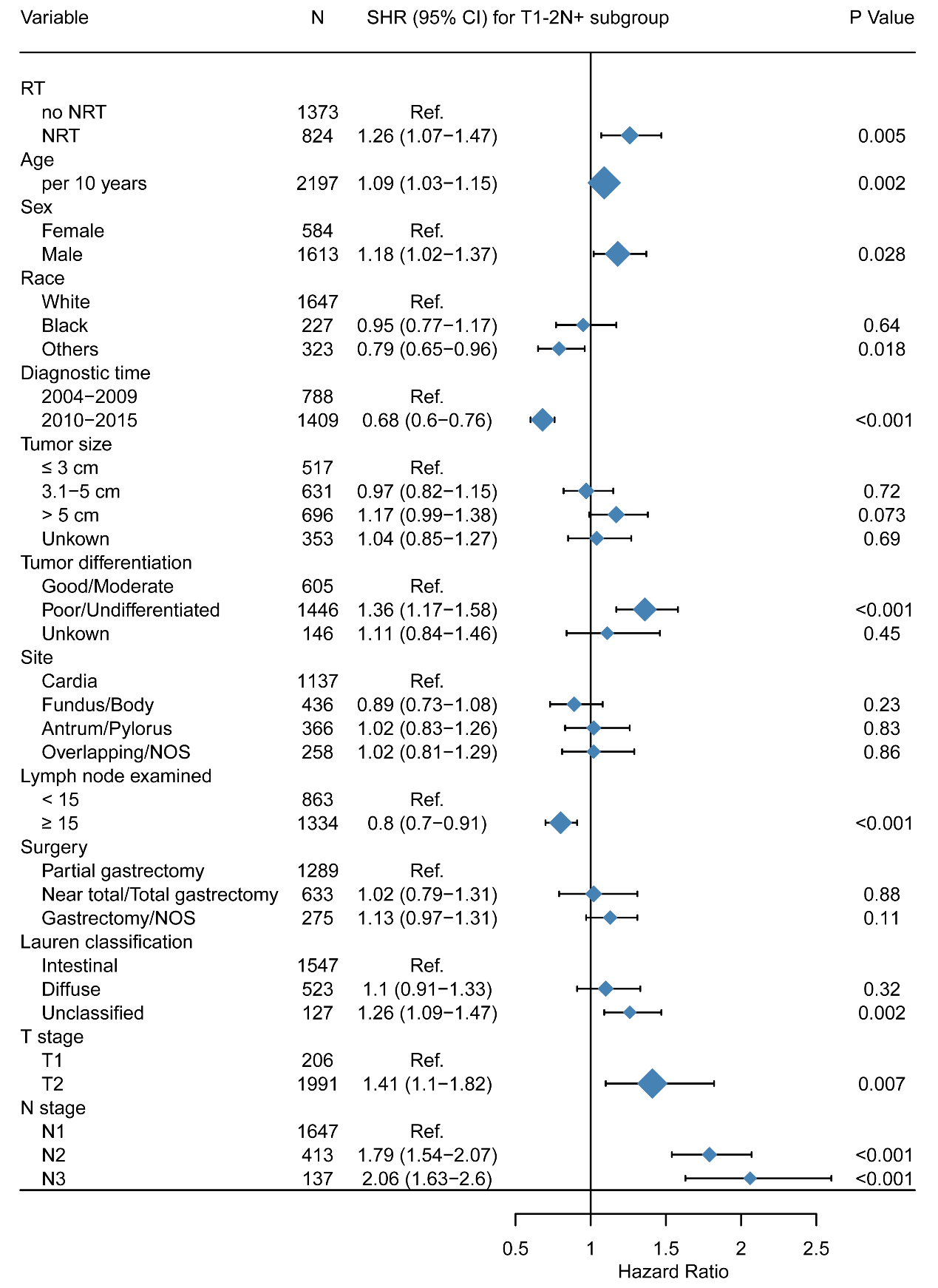


Supplementary Figure 3. Forest plot of competing risk model in T_1-2_N^+^ subgroup.

RT: radiation therapy; NRT: neo-adjuvant radiotherapy; SHR: subdistribution hazard ratio; CI: confidence interval; NOS: not otherwise specific.


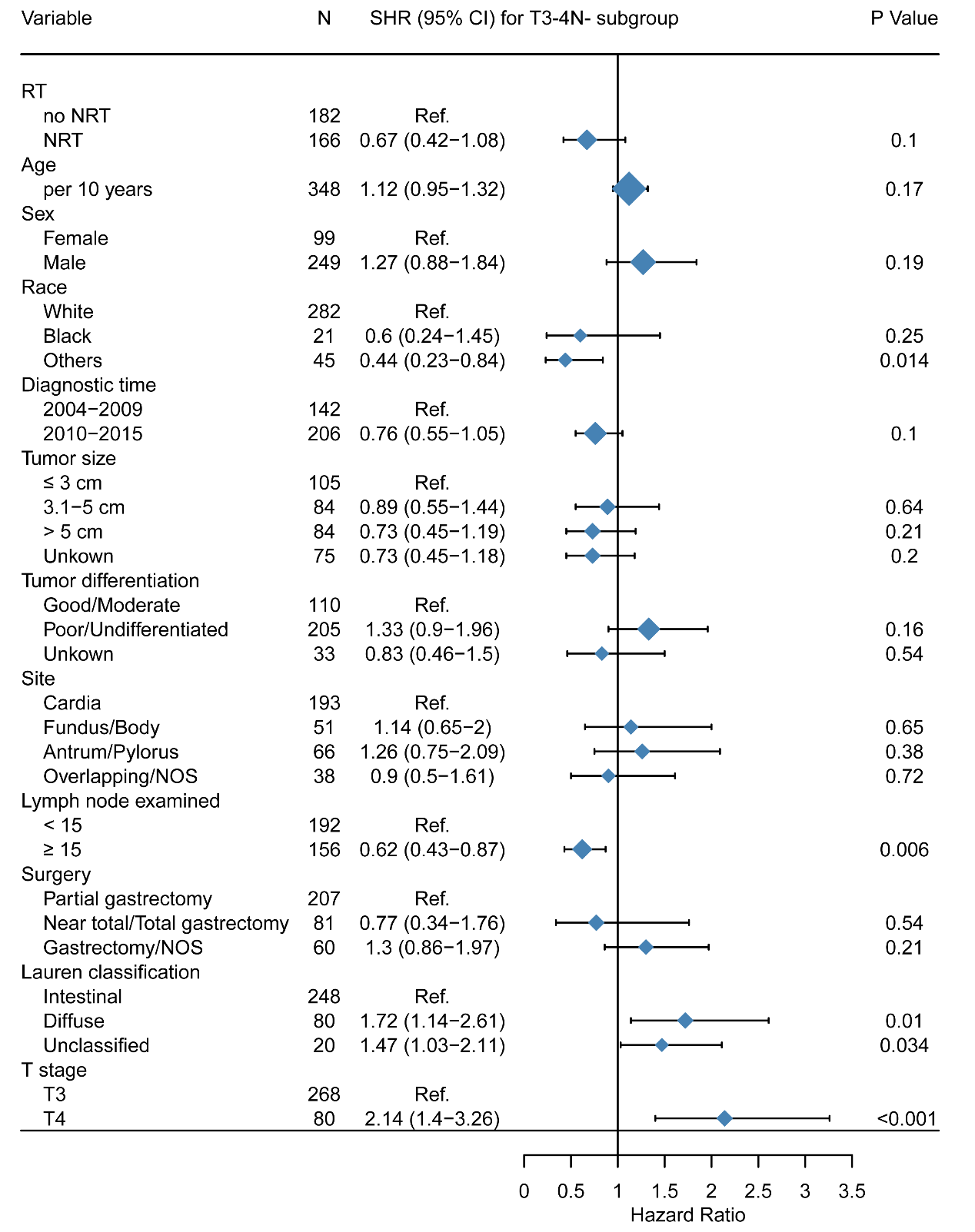


Supplementary Figure 4. Forest plot of competing risk model in T_3-4_N^-^ subgroup.

RT: radiation therapy; NRT: neo-adjuvant radiotherapy; SHR: subdistribution hazard ratio; CI: confidence interval; NOS: not otherwise specific.


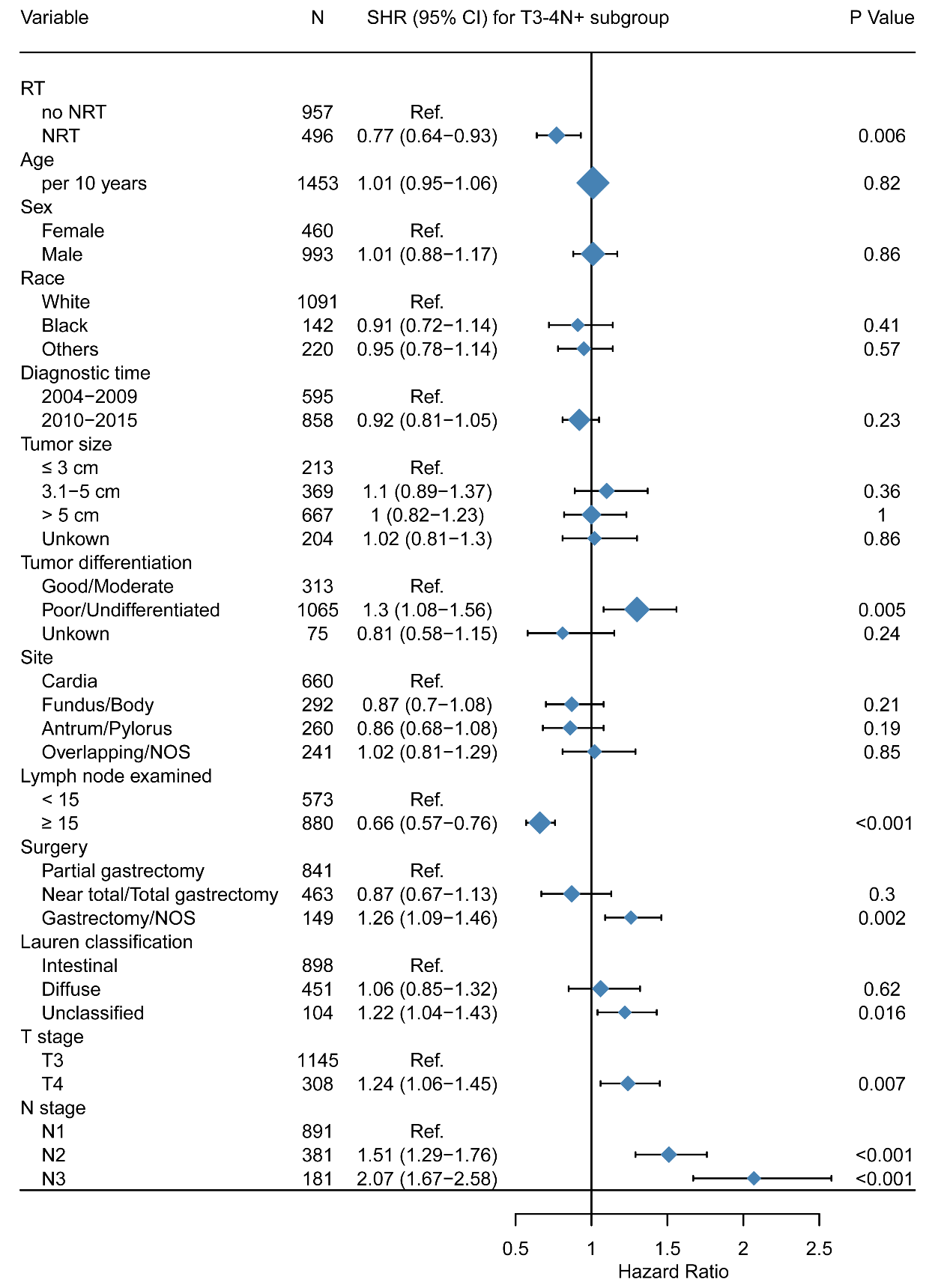


Supplementary Figure 5. Forest plot of competing risk model in T_3-4_N^+^ subgroup.

RT: radiation therapy; NRT: neo-adjuvant radiotherapy; SHR: subdistribution hazard ratio; CI: confidence interval; NOS: not otherwise specific.
